# Bioreactor performance parameters for an industrially-promising methanotroph *Methylomicrobium buryatense* 5GB1

**DOI:** 10.1186/s12934-015-0372-8

**Published:** 2015-11-16

**Authors:** Alexey Gilman, Lieve M. Laurens, Aaron W. Puri, Frances Chu, Philip T. Pienkos, Mary E. Lidstrom

**Affiliations:** Department of Chemical Engineering, University of Washington, Seattle, WA 98195 USA; National Bioenergy Center, National Renewable Energy Laboratory, Golden, CO USA; Department of Microbiology, University of Washington, Seattle, WA 98195 USA

**Keywords:** Methanotroph, Bioreactor, Continuous culture, Methane, Gas-to-liquid

## Abstract

**Background:**

Methane is a feedstock of interest for the future, both from natural gas and from renewable biogas sources. Methanotrophic bacteria have the potential to enable commercial methane bioconversion to value-added products such as fuels and chemicals. A strain of interest for such applications is *Methylomicrobium buryatense* 5GB1, due to its robust growth characteristics. However, to take advantage of the potential of this methanotroph, it is important to generate comprehensive bioreactor-based datasets for different growth conditions to compare bioprocess parameters.

**Results:**

Datasets of growth parameters, gas utilization rates, and products (total biomass, extracted fatty acids, glycogen, excreted acids) were obtained for cultures of *M. buryatense* 5GB1 grown in continuous culture under methane limitation and O_2_ limitation conditions. Additionally, experiments were performed involving unrestricted batch growth conditions with both methane and methanol as substrate. All four growth conditions show significant differences. The most notable changes are the high glycogen content and high formate excretion for cells grown on methanol (batch), and high O_2_:CH_4_ utilization ratio for cells grown under methane limitation.

**Conclusions:**

The results presented here represent the most comprehensive published bioreactor datasets for a gamma-proteobacterial methanotroph. This information shows that metabolism by *M. buryatense* 5GB1 differs significantly for each of the four conditions tested. O_2_ limitation resulted in the lowest relative O_2_ demand and fed-batch growth on methane the highest. Future studies are needed to understand the metabolic basis of these differences. However, these results suggest that both batch and continuous culture conditions have specific advantages, depending on the product of interest.

**Electronic supplementary material:**

The online version of this article (doi:10.1186/s12934-015-0372-8) contains supplementary material, which is available to authorized users.

## Background

An estimated 140 billion cubic meters of natural gas were flared globally in 2011—an equivalent of 5000 trillion BTU [1]. Flaring of natural gas occurs in oil drilling sites around the world [1]. The main difficulties in utilizing this energy for practical applications are factors such as remote locations of the drill sites, the relatively small quantity of natural gas per site, and the limitation of the available chemical catalysis technologies to convert the natural gas to value added products. The remote natural gas sites that fit this description are referred to as stranded natural gas [2]. Investments in a pipeline or gas-to-liquid conversion plant (Fischer–Tropsch process) at these sites are often not profitable due to the high capital expense and the requirement for very high volumes of natural gas to achieve economies of scale [3]. Capturing the energy from stranded natural gas in a cost-effective way would prevent energy waste and may help mitigate greenhouse gas emissions. In addition, biogas is also produced at numerous small sites (sewage treatment plants, landfills, feedlots, etc.) where it is commonly flared, and represents a potentially renewable methane feedstock for the future [4].

**Fig. 1:**
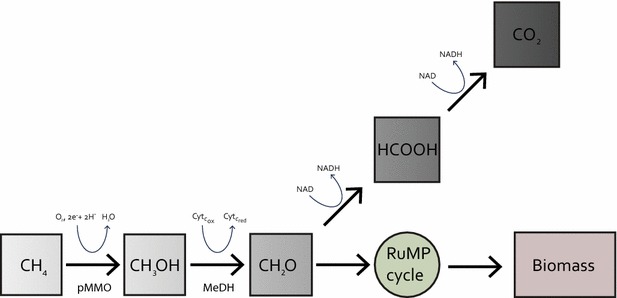
Core metabolism of methane in Group I methanotrophs. *pMMO* particulate methane monooxygenase, *MeDH* methanol dehydrogenase, *RuMP cycle* Ribulose monophosphate cycle

An alternative to catalytic gas-to-liquid conversion technology is biological conversion via methanotrophic bacteria. Methanotrophs are a group of bacteria that utilize methane (the majority component of natural gas and biogas) as their sole carbon and energy source [5, 6]. Unlike chemical catalytic processes, which require high temperature and pressure, methanotrophs convert methane to multicarbon compounds at ambient conditions. A process utilizing methane oxidizing bacteria that does not have the high capital and operations expense associated with high temperature and pressure processes, has the potential to become economically viable in converting stranded natural gas into value added chemicals [7]. *Methylomicrobiumburyatense* 5GB1 has been identified as a promising candidate for use in industrial bio-catalytic methane conversion processes [4, 8, 9]. *M. buryatense* 5GB1 is a gamma-proteobacterial methanotroph utilizing the ribulose monophosphate (RuMP) cycle for formaldehyde assimilation (Fig. 1). In such bacteria, under specific growth conditions, methane is oxidized to methanol by a membrane-bound enzyme called the particulate methane monooxygenase (pMMO). Methanol is subsequently converted to formaldehyde by methanol dehydrogenase. Formaldehyde is then either further oxidized to formate and then to CO_2_ via NAD-linked steps, or converted into multi-carbon intermediates for biomass synthesis via the RuMP cycle (Fig. 1) [5].

*M. buryatense* 5GB1 is a relatively fast-growing methanotroph, with a maximum growth rate of 0.231 h^−1^ (2.9 h doubling time) [9]. It is a moderate haloalkaliphile derived from a parent strain that was originally isolated from a soda lake in Eastern Russia [4]. It grows optimally at 0.75 % w/w NaCl and pH 9.5. This strain is therefore not easily susceptible to contamination and is a good candidate for a scalable process. It is also unusually robust for a methanotroph, and can withstand a relatively wide range of growth conditions. In addition, a suite of genetic tools are available for this strain, and genetic manipulation is relatively facile [9].

Like all gamma-proteobacterial methanotrophs, *M. buryatense* 5GB1 has increased lipid content compared to standard heterotrophs due to the presence of a stacked internal membrane system that houses the pMMO [4, 10, 11]. High lipid content is industrially attractive, as the lipids may be used as a precursor for other products. In addition, acetyl-CoA, the building block for lipids, may be utilized in a synthetic pathway to produce value-added chemicals. The recent discovery of a novel fermentation pathway in gamma-proteobacterial methanotrophs raises the possibility of industrial application for the production of excreted fermentation products [12]. In order to develop this strain as a platform for industrial production of value-added products from methane, a baseline dataset is needed as a starting point for both strain and process development.

In this work, we present a process parameter dataset that was acquired in lab-scale bioreactor experiments under unrestricted (with methane or methanol as the carbon source), methane-limited, and O_2_-limited growth conditions. The dataset includes: maximum specific growth rate, cell lipid content, biomass carbon and nitrogen content, specific methane and specific O_2_ uptake rates, yield as carbon conversion efficiency (CCE), and O_2_/CH_4_ uptake ratios.

## Results and discussion

**Fig. 2:**
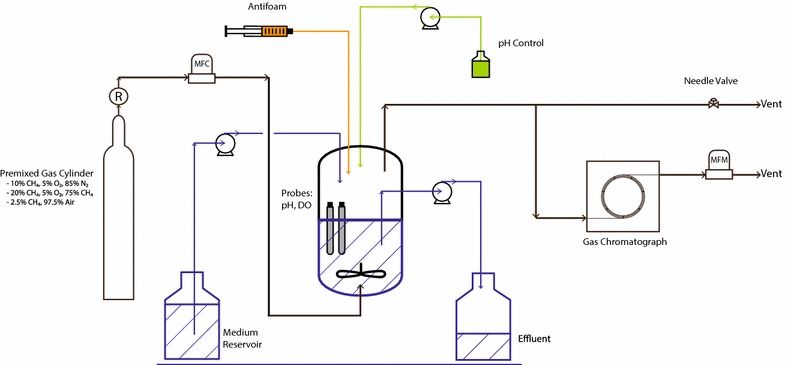
Schematic of bioreactor experimental setup. New Brunswick bioflo310 bioreactor with out-gas sampled by Shimadzu GC2014 gas-chromatograph. The gas flow rate to the GC was maintained constant during the experiment by a needle valve and a mass flow meter (MFM). Inlet gas was supplied via a premixed gas cylinder and the flow rate was regulated by a mass flow controller (MFC). pH was controlled and dissolved O_2_ monitored throughout the experiment. Antifoam was delivered via a syringe pump to prevent foam buildup and base was fed to maintain constant pH

*M. buryatense* 5GB1 was tested in a bench-scale bioreactor (Fig. 2) over four different growth conditions to provide a range for comparison: two as batch culture (grown on methane and methanol) and two as continuous-growth culture (methane-limited, O_2_-limited). In each case, a set of parameters was measured for two replicate bioreactor runs. These parameters include: growth rate, cell dry weight (CDW), methane and O_2_ uptake rates, biomass fatty acids (reported as fatty acid methyl esters, FAME, and assumed to be generated mainly from membrane phospholipids [4, 11]), glycogen as % CDW, and excreted organic acids (formate, acetate, and lactate) (Tables 1, 2). The total biomass carbon and nitrogen contents were measured to be (w/w) 45.0 ± 1.3 and 9.9 ± 1.0 %, respectively, and total excreted organic material that was non-dialyzable was 10.2 ± 0.9 % of the cell dry weight.

**Table 1:** Parameters for unrestricted growth in batch 1L bioreactor

Experiment ID	FM03	FM21	FM18	FM23
Carbon source	Methane	Methane	Methanol	Methanol
Total FAME (% cell dry weight)	8.2	8.5	6.0	5.1
Specific growth rate (h^−1^)	0.239	0.224	0.169	0.173
Cell density (gCDW L^−1^)	0.34	0.31	0.81	0.54
Oxygen uptake (mmol h^−1^)	7.6	7.6	9.1	5.1
Methane uptake (mmol h^−1^)	5.8	6.1	N/A	N/A
Specific oxygen uptake (mmol gCDW^−1^ h^−1^)	22.4	24.7	11.3	9.4
Specific methane uptake (mmol gCDW^−1^ h^−1^)	17.1	19.8	N/A	N/A
O_2_/CH_4_ uptake ratio	1.3	1.2	N/A	N/A
Excreted products				
Formate (μmol gCDW^−1^)	400	672	11,758	13,486
Acetate (μmol gCDW^−1^)	96	123	55	56
Lactate (μmol gCDW^−1^)	26	62	16	31
Glycogen content (% CDW)	N/A	2.7 ± 1.1	N/A	42.8 ± 17.5
Inlet gas composition	10 % CH_4_, 5 % O_2_, 75 % N_2_	10 % CH_4_, 5 % O_2_, 75 %N_2_	0.5 % CH_3_OH^a^, Air	0.5 % CH_3_OH^a^, Air

Summary of process parameters and results for two batch bioreactor experiments using methane and methanol as carbon source. Completed in duplicate

Fed-batch gas uptake rates are at the time of harvest. Secreted products are measured at the time of harvest and represent accumulated concentration

*FAME* Fatty Acid Methyl Ester

^a^Initial methanol concentration by volume. No additional methanol is added during the experiment

**Table 2:** Parameters for continuous growth conditions in 1L bioreactor with substrate limitation

Experiment ID	FM50	FM51	FM38	FM39
Limiting condition	Oxygen limited	Oxygen limited	Methane limited	Methane limited
Total FAME (% cell dry weight)	10.5	10.7	10.2	10.5
Dilution rate (h^−1^)	0.119	0.122	0.122	0.126
Cell density (gCDW L^−1^)	0.79	0.77	0.45	0.46
Dissolved oxygen (% of saturation)	1	1	92	92
Oxygen uptake (mmol h^−1^)	8.8	9.5	6.2	5.9
Methane uptake (mmol h^−1^)	7.7	8.2	3.8	3.6
Specific oxygen uptake (mmol gCDW^−1^ h^−1^)	11.1	12.3	13.7	12.8
Specific methane uptake (mmol gCDW^−1^ h^−1^)	9.7	10.6	8.4	7.8
O_2_/CH_4_ uptake ratio	1.1	1.2	1.6	1.6
Carbon conversion efficiency (%)	46	43	54	61
Excreted products				
Formate (μmol gCDW^−1^)	286	301	255	459
Acetate (μmol gCDW^−1^)	36	85	32	28
Lactate (μmol gCDW^−1^)	24	28	47	21
Glycogen content (% CDW)	7.2 ± 1.9	13.1 ± 4.0	6.0 ± 0.5	8.1 ± 2.5
Inlet gas composition	20 % CH_4_, 5 % O_2_, 75 % N_2_	20 % CH_4_, 5 % O_2_, 75 % N_2_	2.5 % CH_4_, 97.5 % Air	2.5 % CH_4_, 97.5 % Air

Summary of process parameters and results for steady-state bioreactor culture under oxygen limitation and methane limitation. Completed in duplicate

*FAME* fatty acid methyl ester

### Unrestricted batch growth on methane and methanol

**Fig. 3:**
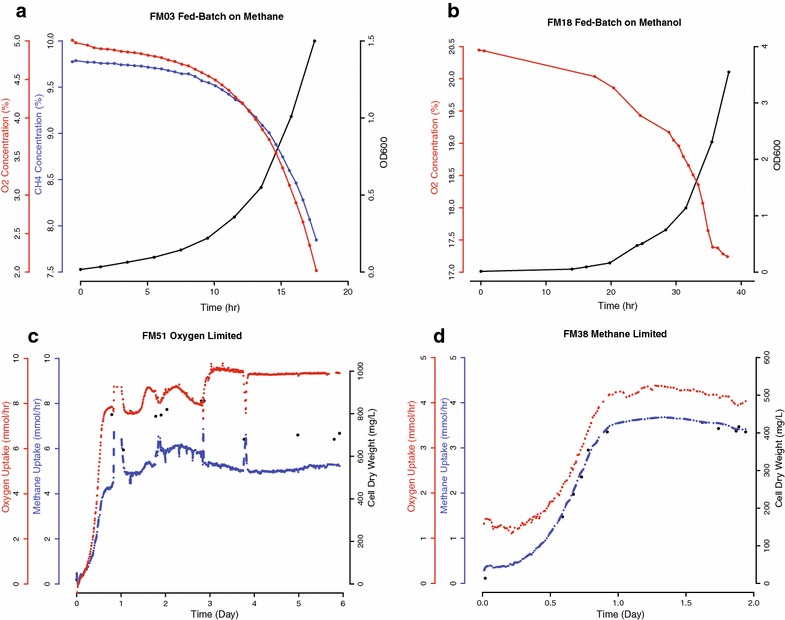
Growth profile and gas uptake data with varying conditions. Gaseous substrate uptake was monitored for batch culture (grown on methane or methanol) and continuous culture (O_2_ or methane-limiting conditions). **a** Unrestricted growth profile with headspace O_2_ and methane concentration. Unrestricted growth rate for *M. buryatense* 5GB1, μ_max_ = 0.224 h^−1^. **b** Unrestricted batch growth on methanol (air was continuously supplied) yielded slower growth compared to methane μ = 0.217 h^−1^. **c** Continuous growth under O_2_ limitation. The culture was supplied with a continuous gas stream at 100 sccm containing 5 % O_2_ and 20 % methane. Dilution rate was set to D = 0.122 h^−1^ and resulted in a stable cell dry weight concentration of ~700 mg L^−1^. **d** Continuous growth under methane limitation. A feed blend of 2.5 % methane in air was supplied at mass flow rate of 100sccm and dilution rate was fixed at D = 0.122 h^−1^. A stable cell density of ~400 mg L^−1^ resulted under these conditions

For unrestricted batch growth, two experimental conditions were compared in duplicate. The first set was carried out as a fed-batch culture with methane as sole carbon source and a second set was carried out using methanol in a batch culture. The experiment was terminated when dissolved O_2_ reached 1 % of saturation to ensure unrestricted growth conditions, which was confirmed by exponential growth (Fig. 3a, b) (Additional files 1, 2). For the methanol experiment, air was continuously supplied to provide excess O_2_. Dissolved O_2_ remained above 85 % of saturation.

*M. buryatense* 5GB1 grew faster on methane compared to methanol; μ_max_ = 0.239 and 0.224 h^−1^ on methane compared with μ_max_ = 0.169 and 0.173 h^−1^ on methanol. On methane, the O_2_/CH_4_ uptake ratios of 1.2 and 1.3 were similar to that predicted from an FBA model for the same growth rate [13].

Methane-grown cultures contained 8.2 and 8.5 % of CDW as extracted fatty acids, and methanol-grown cultures contained 5.1 and 6.0 % of CDW. As the main methane oxidation machinery (pMMO) is housed within the cell membrane, decreased extracted fatty acid content of the cells is in agreement with decreased membranes during methanol growth [14]. In methane-grown cells, the % fatty acid is similar to a previously reported value of 8.1 % for batch culture of *Methylococcus capsulatus* Bath [11] and the fatty acid composition (Additional file 3: Table S1) was similar to that reported previously for a related *M. buryatense* strain [4] and other reported methanotrophic bacteria [15, 16], being dominated by C16 fatty acids, with four different C16:1 positional isomers; C16:1n9, C16:1n7, C16:1n6 and C16:1n5.

When grown on methanol, *M. buryatense* 5GB1 excreted an unusually large amount of formate (as high as 13,486 μmol gCDW^−1^), compared to a maximum of 672 μmol gCDW^−1^ when grown on methane. In addition, methanol-grown cells accumulated a large amount of glycogen (42.8 ± 17.5 % of CDW) compared to 2.7 ± 1.1 % in methane-grown cells. Both the high formate and glycogen have been reported previously for the related strain *M. buryatense* 5B and attributed to unbalanced growth [14, 17]. Glycogen represents a carbon sink that may not be available to generate targeted products and may not be a desirable trait for a commercial strain. Excreted acetate and lactate were low under both conditions, ranging between 16 and 123 μmol gCDW^−1^. For methane-grown cells, the total excreted carbon in formate, acetate, and lactate accounted for 1.8 and 2.9 % of the carbon in biomass, consistent with previous results from a different *Methylomicrobium* species [12].

### Methane limitation

Stable cultures with cell densities of 0.45 and 0.46 g CDW L^−1^ (Fig. 3d) (Additional file 4) were achieved with the dilution rates of 0.122 and 0.126 h^−1^, respectively. The dissolved O_2_ probe maintained a reading of 92 % of saturation with air under these conditions.

Methane limitation resulted in a total fatty acid concentration of 10.2 and 10.5 % of CDW, higher than in the fed-batch cultures. The O_2_/CH_4_ uptake ratio of 1.6 for both replicates is in the range reported for other gammaproteobacterial methanotrophs for methane-limited cultures (1.41–2.15) [18–20], and higher than the fed-batch value. Additionally, the calculated CCE values of 54 and 61 % are in the upper range (42–67 %) of those previously reported for methane-limited culture [18–20].

Excreted formate, acetate, and lactate were shown to be low, at 1.1–1.5 % of the biomass carbon. Formate concentration did not exceed 459 μmol gCDW^−1^ and excreted acetate or lactate concentrations did not exceed 47 μmol gCDW^−1^ (Table 2). Likewise, a moderate amount of glycogen was produced under both methane and O_2_—limiting conditions, 6.0–13.2 % CDW, higher than the fed-batch culture when grown on methane (Table 2).

For process development, the increased O_2_ demand for methane-limited cultures is detrimental due to the potential cost of supplying enriched O_2_, but the higher CCE is attractive since more product can be made per mole of methane used. A full technoeconomic model will be needed to determine whether methane- or O_2_-limitation would be more feasible for a continuous process.

### O_2_ limitation

The dilution rate for O_2_ limitation was set to match methane limitation conditions within experimental error. The observed cell densities of 0.79 and 0.77 g CDW L^−1^ (Fig. 3c) (Additional file 5) are significantly higher than under methane-limiting conditions and are accompanied by a higher specific methane oxidation rate. The dissolved O_2_ probe maintained a reading of ~1 % of saturation during continuous growth. O_2_-limited growth resulted in a fatty acid content (10.5 and 10.7 % of CDW), similar to that of the methane-limited culture. The CCEs of 46 and 43 % (Table 2) are lower than methane limitation, but within the upper range of the values previously reported for other gammaproteobacterial methanotrophs under O_2_-limitation (36–48 %) [18, 19, 21]. The lower CCE values compared to methane limitation suggest that more formaldehyde is oxidized to CO_2_ for NADH production, indicating a higher NADH demand under these growth conditions (Fig. 1). The O_2_/CH_4_ ratios of 1.1 and 1.2 are lower than for the methane-limited cultures and slightly lower than for fed-batch cultures, as might be expected under O_2_-limitation. Since every molecule of methane utilized requires one molecule of O_2_ for the pMMO reaction (Fig. 1), the low ratio indicates a metabolism in which very little of the ATP required for growth is generated from NADH oxidation via oxidative phosphorylation [13]. Further experiments will be required to determine how the metabolic networks change under these conditions, but it is clear that significant metabolic differences occur dependent on the culture conditions.

## Conclusions

*M. buryatense* 5GB1 shows attractive features as a potential bioconversion strain, with both growth rate and carbon conversion efficiency near the top of reported methanotroph values. The datasets presented here provide foundational information for future strain and process development. Such comprehensive datasets are not currently published for any methanotroph. For biomass-derived products, methane-limitation suggests a promising process condition. The resulting culture exhibited the highest CCE and the highest cell membrane content of the tested conditions. However, methane-limitation results in a higher O_2_/CH_4_ uptake ratio and a significant glycogen content. Since a glycogen-negative mutant of *M. buryatense* is now available [9], it may be possible to redirect more carbon into non-glycogen biomass via this strain.

Future process work should focus on high-density fermentation. Cell densities as high as 14 g CDW L^−1^ have been reported for a methanotroph [22], suggesting that with appropriate medium and bioreactor configurations, industrial-scale processes can be developed. Finally, flux analysis and transcriptomics datasets are needed to understand the metabolic differences occurring in each of these growth conditions. The differences noted suggest a metabolic flexibility in *M. buryatense* 5GB1 that should be advantageous for future bioprocess strain manipulation.

## Methods

### Strain handling and cultivating

Strain 5GB1 is a lab variant of wild-type *Methylomicrobiumburyatense* 5G [4]; Cells were grown in NMS2 medium as described in [9]. The inoculum was prepared by growth in a 250 mL serum bottle containing 50 mL medium and incubated for 24 h.

### Bioreactor setup

All the described experiments were completed in a New Brunswick BioFlo310 bioreactor (Eppendorf, Inc. Enfield, CT, USA) as shown in (Fig. 2). A working volume of 1L was used in a 2.5 L vessel. Depending on the experiment, one of the following gas blends was used (vol/vol): 2.5 % CH_4_, 97.5 % air; 10 % CH_4_, 5 % O_2_, 85 % N_2_; 20 % CH_4_, 5 % O_2_, 75 % N_2_. Premixed gas cylinders were purchased from PraxAir (Seattle, WA, USA). The following process parameters were used for all experiments: temperature = 30 °C, agitation: 1000 rpm, gas flow rate = 100 sccm, pH = 8.8. pH was controlled via 2 M NaOH delivered by peristaltic pump from BioFlow310 controller unit.

The bioreactor off-gas line was connected to a gas chromatograph (GC) for continuous gas composition sampling. Shimadzu GC2014 (Shimadzu America, Inc, Columbia, MD, USA) with in-line TCD & FID detectors. Consistent gas flow rate was insured by splitting the off-gas line. The instrument was calibrated with a premixed gas standard at the operation flow-rate of 50 sccm (Restek, Inc, Bellefonte, PA, USA).

### Antifoam

Struktol J 660R (Struktol, Hamburg, Germany) food grade antifoam had been used to control foaming during all continuous experiments. Antifoam was continuously delivered by New Era1000 syringe pump (New Era Pump Systems, Farmingdale, NY, USA) at a flow rate of 20 μL/hr.

### Sampling and OD measurements

Optical density (OD_600_) measurements were conducted at 600 nm wavelength using a JENWAY 7300 (Bibby Scientific, Burlington, NJ, USA) spectrophotometer per manufacturers guidelines. Specific growth rate is determined from the volume of effluent during steady state. Steady-state was defined as a minimum of 3 volume turnovers with a constant OD_600_. All values presented with a ±sign represent a minimum of 3 measurements, with the ±reporting the standard error.

### Gas uptake

Gas uptake was calculated by taking a gas species balance between the inlet and the outlet. The outlet species composition was measured via the GC, and the outlet flow rate during the experiment was determined by completing a N_2_ balance. Specific uptake was calculated by using a conversion factor between *M. buryatense* 5GB1 optical density and cell density (cell dry weight method).

### Lipid analysis

Wet biomass was harvested and centrifuged at 5000 rpm, 20 min. The supernatant was removed and the biomass lyophilized and stored at −80 °C before lipid extraction. Lipid analysis was performed at National Renewable Energy Lab (NREL; Golden, CO) or Matrix Genetics Inc. (Seattle, WA). NREL procedure: 7–10 mg of lyophilized microbial biomass (dried overnight at 40 °C under vacuum) was homogenized with 0.2 mL of chloroform:methanol (2:1 v/v), and the resulting solubilized lipids were transesterified in situ with 0.3 mL of HCl:methanol (5 %, v/v) for 1 h at 85 °C in the presence of tridecanoic acid (C13) methyl ester as an internal standard. FAMEs were extracted with hexane (1 mL) at room temperature for 1 h and analyzed by gas chromatography:flame ionization detection (GC:FID) on an Agilent (Santa Clara, CA, USA) 6890 N with a DB-WAX column with dimensions 30 m × 0.25 mm i.d. and 0.25 μm film thickness (as described in [23]). Individual fatty acids were identified by mass spectrometry for the location of the unsaturation of the 5 individual C16:1 fatty acid positional isomers. The FAMEs were quantified based on a 37-FAME calibration mixture, and each of the positional isomers was quantified based on the response of palmitoleic acid present in the calibration mix (Supelco, certified reference material, CRM47885, Sigma-Aldrich, St. Louis, MO, USA) after normalizing for the internal standard. The sum of the individual fatty acids was calculated and expressed as weight % of dry biomass.

Matrix Genetics Procedure: To 1 or 2 mg of lyophilized samples, 1.5 mL of a 2.5 % sulfuric acid solution in methanol was added in 15 mL sealable glass culture tubes. Samples were sealed tightly with a phenolic cap and sonicated until all material was in solution. Samples were placed in a heatblock and heated at 90 °C for 2 h with mixing, then removed from the heat block and allowed to cool to room temperature. 20 µL of a freshly made 1 mg/mL solution of C13 FAME internal standard in hexanes was added to each vial. 600 µL of hexane and 1.5 mL of 1 % NaCl was added. Samples were vortexed vigorously for 5–10 s, then centrifuged at 4000 rpm for 15 min. A 150 µL partition of the hexane layer from each sample was removed and placed into a GC vial containing a conical glass insert. Agilent 7890-A GC with FID detector and 15 m 123-BD11 column was used (Santa Clara, CA, USA).

### Excreted acids

Formate, acetate, and lactate concentrations in the growth medium were analyzed using NMR spectroscopy by UW-SLU NMR Spectroscopy Resources (UW South Lake Union NMR facility, Seattle, WA, USA) as previously described [12].

### CHN analysis

Biomass percent carbon and nitrogen composition was determined using a Perkin Elmer CHN Analyzer model 2400 (Waltham, Massachusetts, USA) per manufacturers specification. This service was completed by Analytical Service Center, Department of Forestry, University of Washington (Seattle, WA, USA).

### Cell dry weight

Cell dry weight to OD correlation was determined by centrifugation of 150 mL of bioreactor culture in pre-weighed tubes. The culture was centrifuged at 5000 rpm for 20 min, supernatant removed, and remaining biomass lyophilized and reweighed. The conversion factor was then calculated to be 0.201 ± 0.002 g CDW L^−1^ OD^−1^.

### Excreted organic carbon

50 mL of cell culture was centrifuged for 20 min at 5000 rpm. The liquid supernatant was removed, passed through a 0.2 µm filter, and dialyzed for 3 days using Slide-A-Lyzer 3.5 K Dialysis Cassettes (Thermo Scientific, Rockford, IL, USA). The liquid sample was analyzed using Shimadzu TOC-V Analyzer (Shimadzu America, Inc, Columbia, MD, USA) for total organic carbon by Analytical Service Center, Department of Forestry, University of Washington (Seattle, WA, USA).

Carbon content in biomass plus excreted non-dialyzable carbon was used to determine carbon conversion efficiency (CCE), defined as the % of utilized carbon from methane that was converted to biomass plus excreted large molecular weight organic carbon [24].

## Additional files

10.1186/s12934-015-0372-8. 5GB1 fatty acid composition when grown on methane and methanol. *M. buryatense* fatty acid composition.
10.1186/s12934-015-0372-8 FM21 Headspace Gas Composition. Replicate bioreactor experiment of unrestricted growth on methane. Optical density and headspace gas composition.
10.1186/s12934-015-0372-8 FM23 Headspace Gas Composition. Replicate bioreactor experiment of unrestricted growth on methanol. Optical density and headspace gas composition.
10.1186/s12934-015-0372-8 FM39 Cell Density and Gas Uptake. Replicate bioreactor experiment of continues culture under methane limitation. Cell density and specific gas uptake rates.
10.1186/s12934-015-0372-8 FM50 Cell Density and Gas Uptake. Replicate bioreactor experiment of continues culture under oxygen limitation. Cell density and specific gas uptake rates.

## Abbreviations

CDW: cell dry weight
OD_600_: optical density at 600 nm
T_d_: doubling time
CCE: carbon conversion efficiency
GC: gas chromatograph
pMMO: particulate methane monooxygenase
MeDH: methanol dehydrogenase
RuMP cycle: ribulose monophosphate cycle
MFC: mass flow controller
FAME: fatty acid methyl ester
FBA: flux balance analysis
