# Supplementary figures and images for: Bioreactor performance parameters for an industrially-promising methanotroph Methylomicrobium buryatense 5GB1
Source: Microb Cell Fact. 2015 Nov 16;14:182. doi: 10.1186/s12934-015-0372-8 (PMC4647623; doi:10.1186/s12934-015-0372-8)

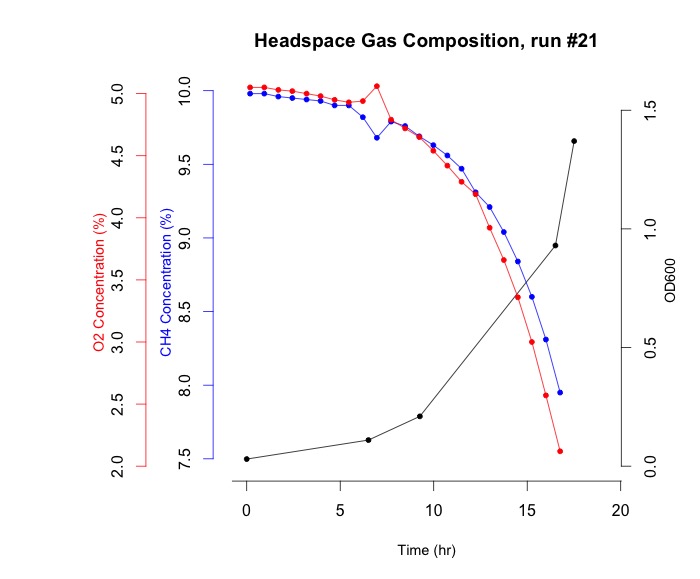

Supplement: Supplementary file 2 — 10.1186/s12934-015-0372-8 FM21 Headspace Gas Composition. Replicate bioreactor experiment of unrestricted growth on methane. Optical density and headspace gas composition. [file 12934_2015_372_MOESM2_ESM.jpeg]

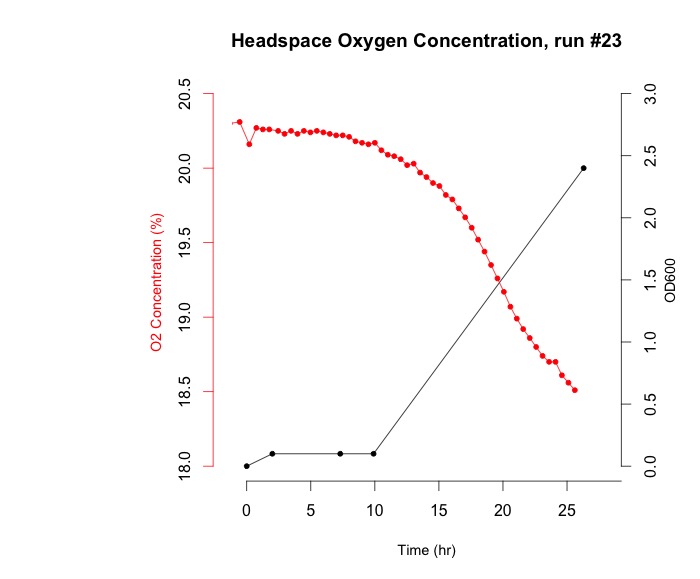

Supplement: Supplementary file 3 — 10.1186/s12934-015-0372-8 FM23 Headspace Gas Composition. Replicate bioreactor experiment of unrestricted growth on methanol. Optical density and headspace gas composition. [file 12934_2015_372_MOESM3_ESM.jpeg]

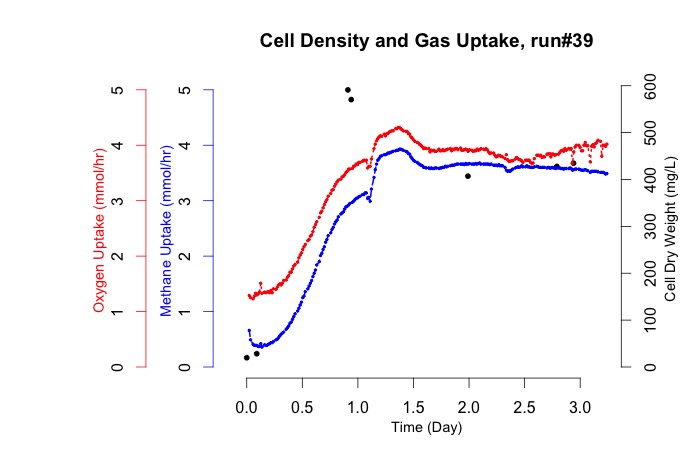

Supplement: Supplementary file 4 — 10.1186/s12934-015-0372-8 FM39 Cell Density and Gas Uptake. Replicate bioreactor experiment of continues culture under methane limitation. Cell density and specific gas uptake rates. [file 12934_2015_372_MOESM4_ESM.jpeg]

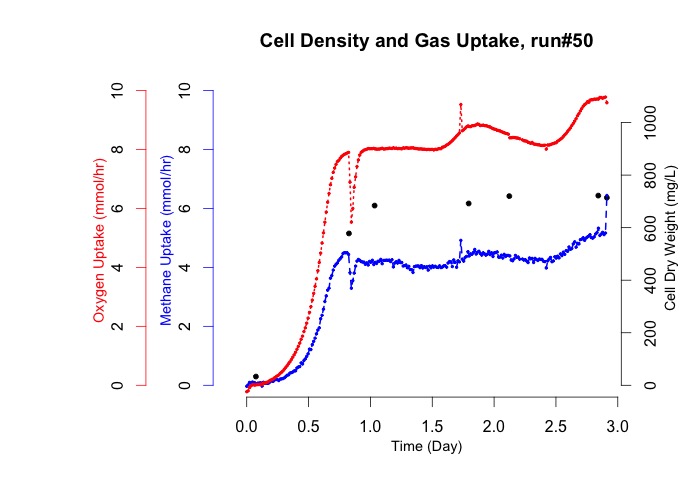

Supplement: Supplementary file 5 — 10.1186/s12934-015-0372-8 FM50 Cell Density and Gas Uptake. Replicate bioreactor experiment of continues culture under oxygen limitation. Cell density and specific gas uptake rates. [file 12934_2015_372_MOESM5_ESM.jpeg]
